# An MSA-P patient presenting with preserved glucose metabolism in the putamen, cerebellar hypometabolism and pronounced loss of presynaptic dopamine transporter in the striatum

**DOI:** 10.1186/s41824-026-00294-8

**Published:** 2026-03-20

**Authors:** Swen Hesse, Manja Schiefer, Solveig Tiepolt, Dorit Prochnow, Larissa Mämecke, Frank Hoffmann, Iñaki Schniewind, Osama Sabri, Björn Falkenburger, Sebastian Brock

**Affiliations:** 1 Department of Nuclear Medicine, University Medical Center, Leipzig, Germany; 2 Department of Neurology, Hospital Martha-Maria Halle, Halle/Saale, Germany; 3 Institute for Radiology and Neuroradiology, Hospital Martha-Maria Halle, Halle/Saale, Germany; 4 MVZ Mitteldeutscher Praxisverbund Humangenetik, Praxis Halle am St. Elisabeth Krankenhaus, Halle/Saale, Germany; 5 German Center for Neurodegenerative Diseases (DZNE), Dresden, Germany; 6 Department of Neurology, University Hospital Carl Gustav Carus, TUD Dresden University of Technology, Dresden, Germany

**Keywords:** SPECT, PET, Dopamine transporter, FDG, [^18^F]fluorodeoxyglucose, Parkinsonian syndrome, Multiple system atrophy, MSA, SPG, Putamen

## Abstract

Positron emission tomography (PET) of the brain using [^18^F]fluorodeoxyglucose (FDG) is becoming increasingly important for the diagnosis and differential diagnosis of atypical parkinsonian syndrome such as multiple system atrophy (MSA), which is characterized by hypometabolism of the putamen, pons, and cerebellum. We report on a patient with clinically established MSA based on a rapidly progressive, poorly levodopa-responsive parkinsonian syndrome, multidomain autonomic failure, and imaging findings where hereditary spastic paraplegia was discussed as a differential diagnosis. PET images revealed a well-preserved glucose metabolism in the striatum, specifically in the putamen, while metabolism in the cerebellum was significantly reduced. This pattern of glucose metabolism might indicate a distinct subtype of synucleinopathy as proven by seed-amplification assay and should be taken into account when diagnosing patients with MSA.

## Interesting image

Multiple system atrophy (MSA) is a rare, adult-onset, progressive neurodegenerative disorder with major diagnostic challenges, specifically in the early phase of the disease, with two primary types, an MSA-parkinsonian type (MSA-P) and an MSA-cerebellar type (MSA-C) depending on the predominant motor phenotype (Poewe et al. 2022).

**Fig. 1:**
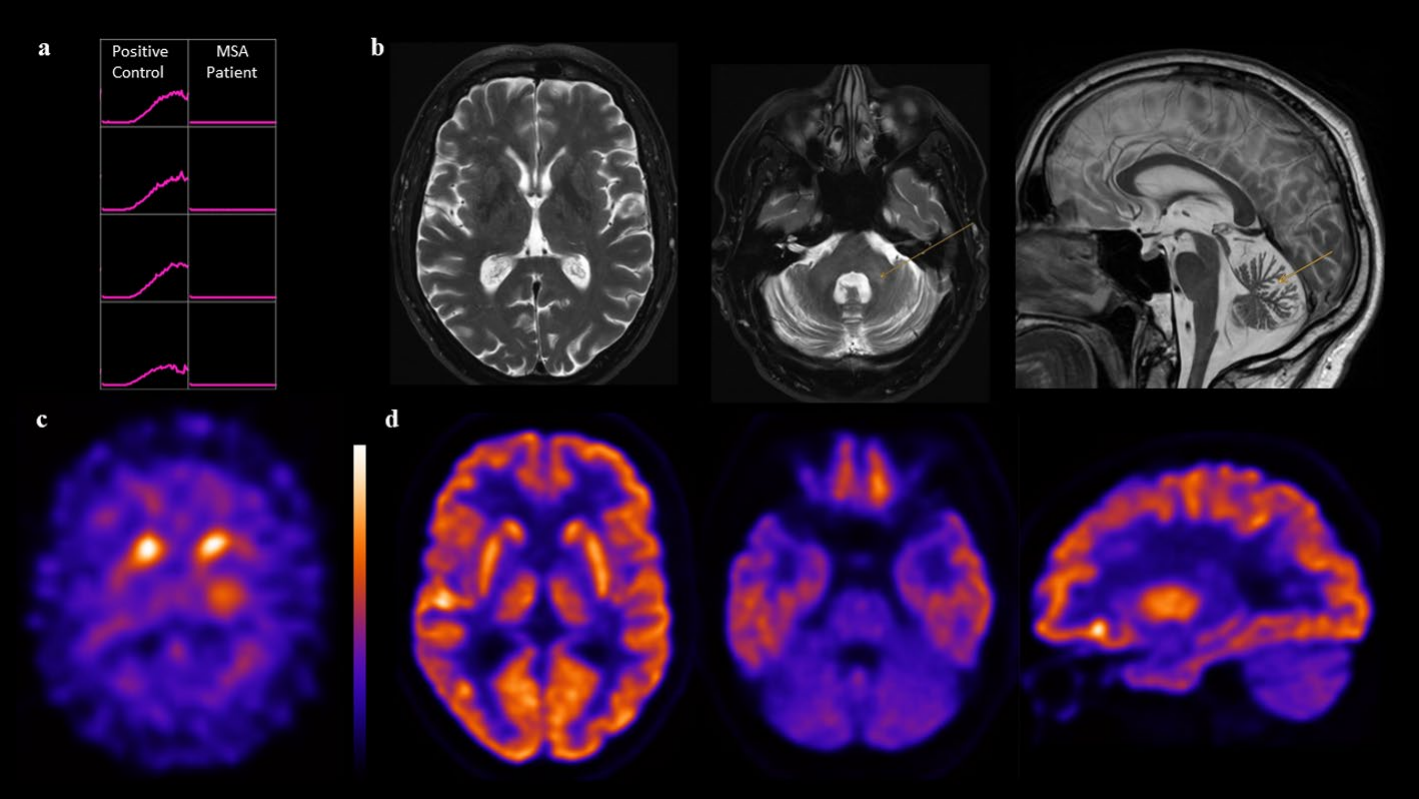
**a** Lewy‑fold‑specific α‑synuclein seed amplification assay (SAA) of cerebrospinal fluid (CSF) shows no detectable seeding activity in the MSA patient compared to a positive control (Parkinson’s disease). Measurements were performed in four replicates. **b** T2 images showing hyperintensity in the pontocerebellar tract and cerebellar atrophy (arrows, transaxial and sagittal slices) but normal putaminal signal (transaxial slice). **c** [^123^I]ioflupane SPECT imaging of the dopamine transporter (DAT SPECT) demonstrate severe nigrostriatal dopaminergic nerve cell loss on both sides with higher reduction in the putamen as compared to the head of the caudate (transaxial slice at striatal level) and **d** [^18^F]fluorodeoxyglucose (FDG) PET images (transaxial slices at striatal and cerebellar level, as well as sagittal slice at midline reveal a disease-specific uptake pattern characteristic of atypical parkinsonism primarily resembling MSA-C, but lacking the more specific feature of loss of putaminal FDG uptake indicative for MSA-P. Imaging was performed at the time of initial diagnosis (MRI) and one year later during the course of the disease (SPECT, PET), while SAA was performed consecutively to support the diagnosis of MSA

This 46-year-old male patient presented with severe autonomic failure (bladder dysfunction, orthostatic hypotension), a very prominent extrapyramidal-motor syndrome and a history of rapid eye movement (REM) sleep behaviour disorder but also bilateral lower-limb gait spasticity. The patient fulfilled clinical criteria of clinically established MSA (Wenning et al. 2022) with poor levodopa responsiveness, symmetric parkinsonism with moderate postural instability and severe hypophonic speech impairment, moderate gait ataxia, and loss of smooth pursuit. The patient exhibited signs of autonomic dysfunction including urinary urge incontinence, post-void urinary residual volume of 370 ml and blood pressure drop of 28/17 mmHg during orthostatic testing. Since early-onset bilateral parkinsonism together with ataxia and stiffness of lower limbs can be associated with hereditary spastic paraplegias (HSP/SPG) as a differential diagnosis (which was further supported by the additional positive Babinski sign on both sides), genetic testing was performed. This testing identified a pathogenic mutation in the SPG4 gene so that HSP type 4 was discussed as an alternative aetiology. An MRI showed cerebellar atrophy and hyperintensity in the middle cerebellar peduncle on T2 images (Fig. 1b). Clinical symptoms progressed rapidly over 2 years and showed no response to high-dose levodopa treatment (1000 mg daily).

Dopamine transporter (DAT) imaging using [^123^I]ioflupane and SPECT revealed a symmetric and severe reduction of DAT in both striata most pronounced in the putamina relative to the caudate head (Fig. 1c). A subsequent [^18^F]fluorodeoxyglucose (FDG) PET showed reduced uptake in the cerebellum (consistent with the MRI and suggestive for MSA) while uptake in the striatum, specifically the putamen bilaterally, was normal (Fig. 1d).

This is an interesting finding since ataxia was only mild. However, previous ‘biomarker-supported’ definition in the development process of the MSA criteria postulate that MSA without ataxia may require [^18^F]FDG PET abnormality in the cerebellum, and MSA without parkinsonism may require hypometabolism in the striatum similar to MRI marker for MSA-P or MSA-C (Wenning et al. 2022).

Thus, when interpreting the scans, nuclear medicine physicians should carefully evaluate both regions to detect subtle hypometabolism in the clinically unaffected brain regions for the diagnosis of MSA. This case represents an unusual presentation of an MSA pattern, as the putamen – one key region – appeared entirely unremarkable on both PET and MRI, despite reduced DAT binding, while the cerebellum – the other key region – showed abnormalities on both modalities.

The newly established α-synuclein seed amplification assays (SAA) to detect α-synuclein pathology in cerebrospinal fluid (CSF) of patients is a highly sensitive and specific test for diagnosis of MSA (Ma et al. 2024; Rossi et al. 2020; Bräuer et al. 2025). The SAA from this patient’s CSF was negative according to the SAA protocol used in this study (Rossi et al. 2020) (Fig. 1a). Although the negative SAA result is in agreement with a diagnosis of MSA, non-synucleinopathies like SPG cannot be ruled out. Clinically, the patient showed rapid progress towards palliative, intensive care, which is in line that MSA patients with severe symptomatic autonomic failure (symptomatic orthostatic hypotension, urinary incontinence, or both) at diagnosis are associated with poor prognosis (Low et al. 2015). Finally, although in cases with SPG, juvenile or early-onset parkinsonism with variable levodopa responsiveness have been reported, particularly in SPG7 and SPG11 (Pedroso et al. 2023), it is unlikely that SPG with striatonigral degeneration alone would explain the rapid course of the disease. SPG4, rarely present with parkinsonism, however, ataxia accompanied by cerebellar atrophy and significantly reduced cerebellar blood flow can occur (Varghaei et al. 2022; Nielsen et al. 2004). Overall, SPECT or PET data on SPG4 are extremely rare.

## Conclusion

In summary, this case shows that MSA is a clinical phenotype that can be pathologically heterogeneous. Here, clinical and biomarker-based findings support the diagnosis of MSA while genetic analysis indicate that SPG4 gene mutation can be associated with an MSA-like phenotype such as leucine-rich repeat kinase 2 (LRRK2) gene mutation plays a role in the pathogenesis of Parkinson’s disease. The extent to which the SPG mutation contributes to the unusual SPECT and PET findings and the very rapid progression of MSA is a matter of debate.

## Data Availability

The data that support the findings of this study are upon reasonable request to the corresponding author at swen.hesse@medizin.uni-leipzig.de.
